# A Dual-Passband Frequency Selective Surface with High Angular Stability and Polarization Insensitivity

**DOI:** 10.3390/mi15060690

**Published:** 2024-05-24

**Authors:** Yi Li, Yan Ma, Peng Ren, Minrui Wang, Zheng Xiang

**Affiliations:** The State Key Laboratory of Integrated Services Networks, Xidian University, Xi’an 710071, China; 22011210589@stu.xidian.edu.cn (Y.M.); pren@xidian.edu.cn (P.R.); mrwang614@163.com (M.W.); zhx@mail.xidian.edu.cn (Z.X.)

**Keywords:** frequency selective surface (FSS), dual passband, high angular stability, polarization insensitivity

## Abstract

In this paper, a dual-passband frequency selective surface (FSS) with high angular stability and polarization insensitivity is proposed. The unit structure consists of a circular aperture, two annular apertures and four cross apertures. The designed FSS can achieve a double-passband at the interested frequencies of 8.45 GHz and 12.76 GHz with an insertion loss of less than 1 dB, and it can retain a stable transmission characteristic with the incident angle ranging from 0° to 86° for TE mode and from 0° to 83° for TM mode. Good agreement between the experimental results and the simulated response verifies the feasibility of the proposed FSS.

## 1. Introduction

Frequency selective surface (FSS) has garnered significant attention over the past few decades [1]. FSS, serving as a spatial filter, finds extensive applications in diverse fields, including antenna design, electromagnetic shielding, and microwave and millimeter-wave devices [2,3,4,5]. Notably, the radar stealth capabilities of the FSS have been a subject of considerable interest [6]. In order to diminish the radar cross-section (RCS) and improve radar stealth effectiveness, specialized FSS designs have been proposed to address variations in incident angles [7]. Changes in incidence angle or polarization state typically lead to alterations in the transmission characteristics of the FSS structure, thereby impacting signal transmission performance. Consequently, researchers are increasingly focusing on the development of FSS that exhibit improved angular stability and reduced sensitivity to polarization.

In recent years, numerous methods have been proposed to improve and enhance angular stability and polarization insensitivity [8,9,10,11,12,13,14,15,16,17,18]. For example, Lee et al. proposed a 3D-shaped FSS that utilizes through-holes in a multilayer printed circuit board structure to achieve a stable frequency response within an incident angle range of 0° to 60° [8]. Similarly, Zhao et al. proposed a quasi-fractal strip structure. This structure comprises a ‘swastika’-shaped metal strip surrounded by four rotationally symmetrical ‘H’-shaped metal strips, which achieve angular stability up to 80° [10]. As spectral resources become increasingly scarce, the need for dual-band capabilities in applications such as modern communication systems and radar technologies grows. Single-band solutions are inadequate, especially in situations requiring high angular stability and polarization insensitivity.

Due to the growing demand for multi-frequency applications, researchers have conducted relevant investigations [15,19,20,21]. For instance, Venkatesh et al. designed a dual-band-stop FSS where the actual measurement results of angular stability reached 60° [15]. Kumar et al. presented a triband band-stop FSS with a stable frequency response up to 60° incidence angle for both TE and TM polarizations [21]. Although some simulated results achieve an angle stability of up to 80°, the actual measured outcomes do not meet the simulated results.

In recent studies, various aspects of dual-band FSS have been explored to enhance performance. Alwahishi et al., focusing on 6G technology, proposed a reconfigurable design that targets the 28 GHz and 38 GHz bands to improve spectrum management and frequency selection [22]. Additionally, they introduced an intelligent FSS unit that optimizes spectrum utilization and system performance, demonstrating significant advancements for future communication systems [23]. Furthermore, their other research investigated a design that allows for adjustable frequency responses through structural modifications, providing flexibility in application [24]. Complementing these studies, Dicandia and Genovesi designed a transmission-type polarization-insensitive and angularly stable polarization rotator using the characteristic modes theory, offering significant advancements in achieving polarization insensitivity and angular stability [25].

In this paper, a dual-passband FSS with high angular stability is proposed, which can achieve angular stability above 83° in TE and TM polarization only by the metal ring gap and the Jerusalem cross. The passbands are located in the X-band and Ku-band, respectively. The organization of this paper is as follows: Section 2 includes a detailed introduction to the unit structure and design process of the FSS, supplemented by an equivalent circuit model (ECM) to elucidate the working principles of the FSS. Section 3 covers simulations of the proposed FSS, including comparisons with the related circuit model. Section 4 details the production process of the FSS prototype and the experimental results obtained, and Section 5 concludes the article.

## 2. Design and Analysis of FSS

### 2.1. Structure Description

**Figure 1:**
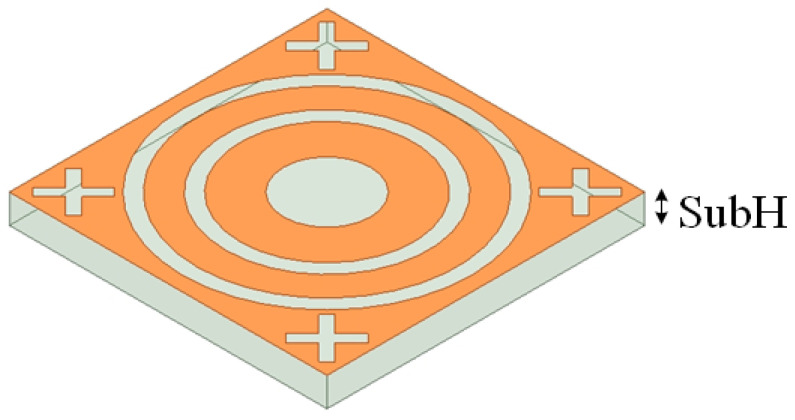
Schematic diagram of FSS unit structure.

The proposed FSS structural design is based on a periodic unit structure, as shown in Figure 1. The unit structure primarily consists of a metallic plate and a dielectric substrate. The metallic plate adopts a gap-type design and covers the upper layer of the dielectric substrate.

**Figure 2:**
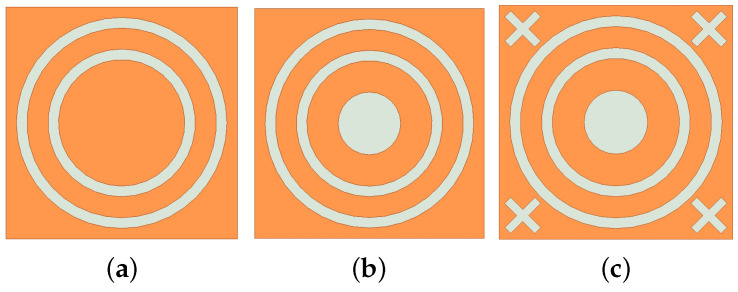
Evolution of proposed FSS unit. (**a**) Stage 1. (**b**) Stage 2. (**c**) Proposed unit.

The construction is based on the double square loop (DSL) structure, which is gradually assembled by joining double circular ring slots. The formation process of the unit cell is illustrated step by step in Figure 2.

**Figure 3:**
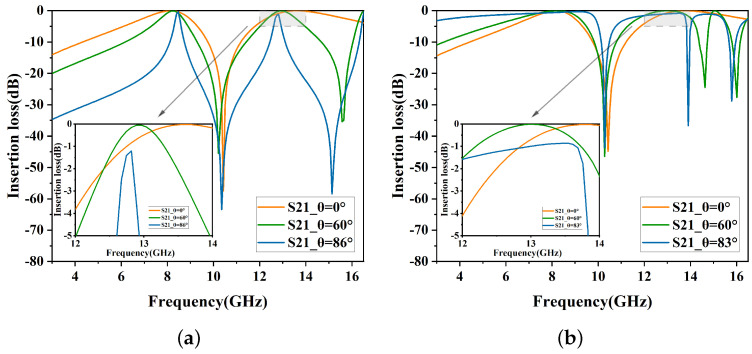
Insertion loss of Stage 1. (**a**) TE polarization. (**b**) TM polarization.

In the initial stage, a dual-band structure is designed according to the DSL specifications, as shown in Figure 2a. The performance is depicted in Figure 3. As the incident angle increases, a partial shift in the resonance frequency of the second band can be observed. For TE polarization, the center frequency of the second band is 13.5 GHz at a 0° incident angle. When the incident angle reaches 86°, the center frequency shifts left to 12.76 GHz. For TM polarization, the center frequency of the second band is 13.9 GHz at a 0° incident angle, which significantly differs from that of TE polarization.

**Figure 4:**
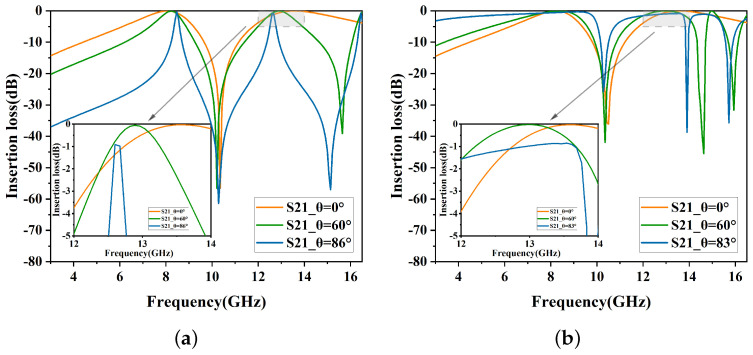
Insertion loss of Stage 2. (**a**) TE polarization. (**b**) TM polarization.

To enhance polarization insensitivity, the second stage incorporates circular slots symmetrically positioned at the center, as shown in Figure 2b. As indicated in Figure 4, for TM polarization, the center frequency of the second band shifts from 13.9 GHz to 13.5 GHz, aligning with the TE polarization. This reduces resonance frequency shifting and marginally improves angular stability.

**Figure 5:**
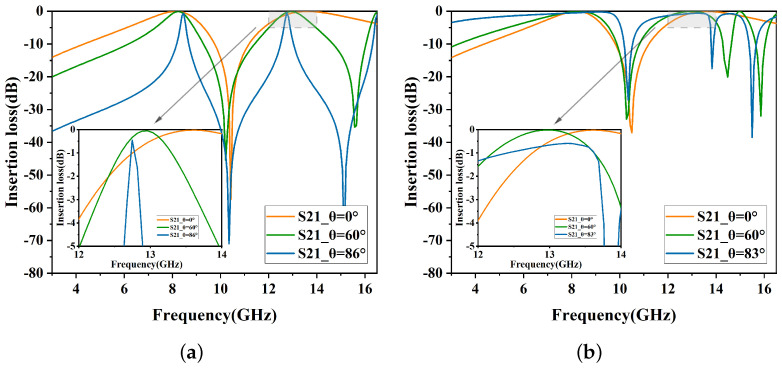
Insertion loss of proposed unit. (**a**) TE polarization. (**b**) TM polarization.

Furthermore, reducing unit cell spacing improves the angular stability of the FSS. The double circular ring structure remains the primary configuration for maintaining stability. Therefore, lattice spacing is adjusted appropriately without affecting the double-circular rings. In the third stage, four centrally symmetric cross-shaped slots are incorporated around the circular ring slots, as shown in Figure 2c. As demonstrated in Figure 5, for TE polarization, the insertion loss decreases from 1.2 dB to 0.5 dB at an incident angle of 86°. For TM polarization, the insertion loss decreases from 1.0 dB to 0.8 dB at an incident angle of 83°. The insertion loss for both TE and TM polarizations is reduced, improving angular stability.

These adjustments and optimized designs ensure that the FSS maintains good performance under different incident angles and polarization conditions, significantly enhancing its angular and polarization stability.

**Figure 6:**
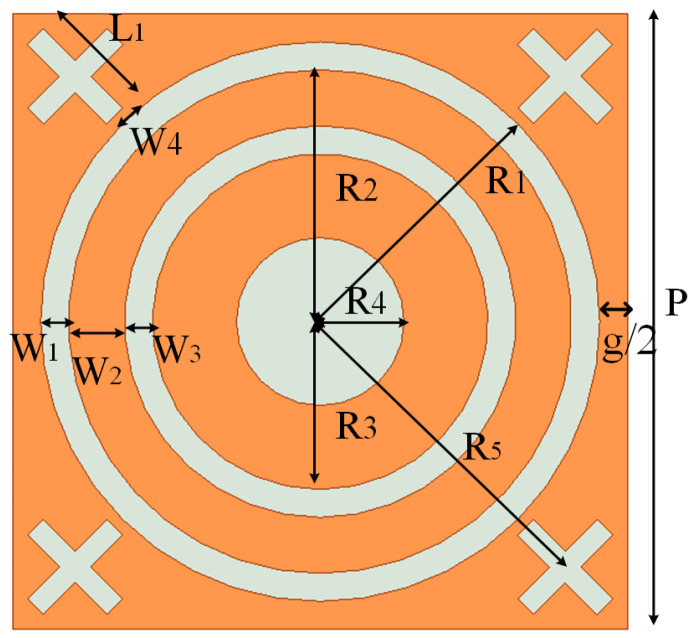
Parameter diagram of FSS unit structure.

The dielectric substrate is arranged in a quadrilateral periodic pattern. The results of the analysis indicate that the quadrilateral exhibits better resonance stability for the TE and TM modes than the triangle design. The dielectric substrate is of a duroid material with a relative dielectric constant of 2.2 and a loss tangent of 0.0009. Copper cladding is applied to the dielectric substrate with a thickness ranging from 0.017 mm to 0.035 mm. The unit cell is shown in Figure 6, and the optimized dimensions of the proposed structure for obtaining the desired response are given in Table 1.

### 2.2. Operation Principle

**Figure 7:**
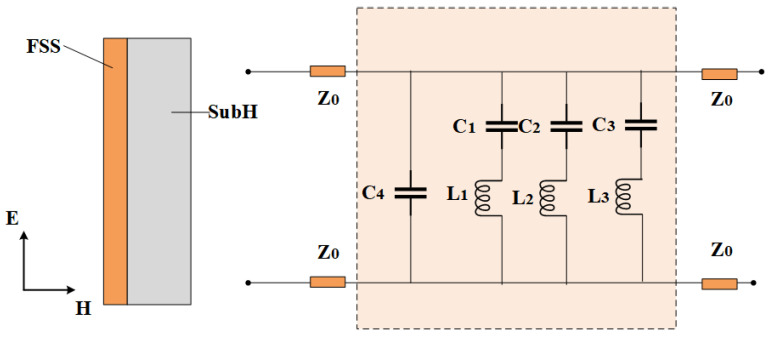
ECM of the proposed FSS. $L_{1}$ = 1.043 nH, $L_{2}$ = 0.028 nH, $L_{3}$ = 0.358 nH, $C_{1}$ = 0.221 pH, $C_{2}$ = 0.165 pH, $C_{3}$ = 10.000 pH, $C_{4}$ = 0.561 pF.

Utilizing the theoretical derivations from traditional filters such as the DSL and gridded square loop (GSL), the equivalent circuit of the unit cell of the proposed FSS is shown in Figure 7. The equivalent circuit representation of the double circular ring patch consists of two shunt serial LC resonators. The values of inductance (L) and capacitance (C) in these LC circuits are influenced by the spacing between conductive elements and components. Lee et al. have summarized the normalized equations for calculating the inductance and capacitance of strip gratings [26], as follows.

(1) $$\begin{array}{cc} X_{\mathrm{TE}} & =F(p,w,\lambda ,\theta )\\ \; & =\frac{p\cos\theta }{\lambda }\left[\ln\left(\csc\frac{\pi w}{2p}\right)+G\left(p,w,\lambda ,\theta \right)\right] \end{array}$$

(2) $$\begin{array}{cc} B_{\mathrm{TM}} & =4F(p,g,\lambda ,\varphi )\\ \; & =\frac{4p\cos\varphi }{\lambda }\left[\ln\left(\csc\frac{\pi g}{2p}\right)+G\left(p,g,\lambda ,\varphi \right)\right] \end{array}$$

(3) $$\begin{array}{c} X_{\mathrm{TM}}=\frac{p\sec\varphi }{\lambda }\left[\ln\left(\csc\frac{\pi w}{2p}\right)+G\left(p,w,\lambda ,\varphi \right)\right] \end{array}$$

(4) $$\begin{array}{c} B_{\mathrm{TE}}=\frac{4p\sec\theta }{\lambda }\left[\ln\left(\csc\frac{\pi g}{2p}\right)+G\left(p,g,\lambda ,\theta \right)\right] \end{array}$$

where *G* is the specified correction term, *p* is the period, *w* is the width of the metal strip, and *g* is the distance between two adjacent rings. θ and φ are the incidence angles, and λ is the incident wavelength. Therefore, following the equivalent circuit for square ring apertures, the ECM for circular apertures in this structure can be derived. The formula for calculating the normalized inductance component of circular ring apertures is provided.

(5) $$\begin{array}{c} L_{1}=\frac{1}{2\pi f}\left(X_{L1\_2}+\frac{w_{1}}{2R_{2}+g+2M}X_{L1\_1}\right) \end{array}$$

(6) $$\begin{array}{c} L_{2}=\frac{1}{2\pi f}\left(\frac{1}{10}X_{L2\_1}+\frac{w_{3}}{2R_{3}+g+2M}X_{L1\_2}\right) \end{array}$$

(7) $$\begin{array}{c} L_{3}=\frac{1}{2\pi f}\left\{\frac{2\pi (R_{3}-R_{4})}{2p}F\left(p,2R_{3}-2R_{4},\lambda ,\theta \right)\right\} \end{array}$$

where

(8) $$\begin{array}{c} X_{L1\_1}=F\left(p,g+2M,\lambda ,\theta \right) \end{array}$$

(9) $$\begin{array}{c} X_{L1\_2}=\frac{\pi R_{2}}{p}F\left(p,2w_{2},\lambda ,\theta \right) \end{array}$$

(10) $$\begin{array}{c} X_{L2\_1}=\frac{\pi R_{3}}{p}F\left(p,2R_{3},\lambda ,\theta \right) \end{array}$$

In the above formulas, *p* denotes the structural period. In Equation (1), which calculates the inductance of the strip metal, *w* represents the width of the strip metal. In Equation (5), *w* is defined as M+2g, indicating the total width of the outermost metal, where *M* represents the average width of the circular gaps. Since the outer metal is circular, the width of the metal varies at every point. In this case, the average of a function formula is employed.

(11) $$\begin{array}{c} f_{\mathrm{avg}}=\frac{\int_{a}^{b}f\left(x\right)dx}{b-a} \end{array}$$

The average width of the outer metal is M+2g.

(12) $$\begin{array}{c} M=\frac{\int_{-d/2}^{d/2}\left[\left(d/2\right)-\sqrt{{\left(d/2\right)}^{2}-x^{2}}\right]dx}{d} \end{array}$$

The branch ($L_{1}-C_{1}$) represents the impedance of the outermost metal conductor containing cross-shaped slots. The branch ($L_{2}-C_{2}$) refers to the impedance of the metal ring formed in relation to the conductor *g* and the width of the circular ring $W_{2}$. The branch ($L_{3}-C_{3}$) represents the impedance formed by the interaction between the conductor of the circular ring with width $W_{2}$ and the innermost metal ring. Due to the parameters of capacitance and inductance being influenced not just by individual slots or conductors, an intermediate variable is introduced. The calculation process for capacitance is as follows:

(13) $$\begin{array}{c} C_{1}=\frac{1}{2\pi f}\varepsilon_{eff}\times \left[F\left(p,2R_{1}-2M,\lambda ,\varphi \right)+F\left(2R_{1}-w_{1},w_{1},\lambda ,\varphi \right)\right] \end{array}$$

(14) $$\begin{array}{c} C_{2}=\frac{1}{2\pi f}\varepsilon_{eff}\times \left[F\left(2R_{1}-w_{1},w_{1},\lambda ,\varphi \right)+F\left(2R_{3},w_{3},\lambda ,\varphi \right)\right] \end{array}$$

In (13) and (14), $\varepsilon_{eff}$ represents the effective relative permittivity of the dielectric substrate. If one side of the FSS contains a thick dielectric substrate, the equivalent capacitance increases by a coefficient of $\varepsilon_{eq}=\left(\varepsilon_{r}+1/2\right)$. For a thin dielectric substrate, the equivalent capacitance is a function of the substrate thickness and permittivity, and its normalized equation is derived:

(15) $$\begin{array}{c} \varepsilon_{eq}=\varepsilon_{r}+\left(\varepsilon_{r}-1\right)\times \left(\frac{-1}{{\left(e^{x}\right)}^{N}}\right);x=\frac{10h}{p} \end{array}$$

where *h* denotes the thickness of the dielectric substrate, and *N* is the exponential factor related to the unit shape, ranging from 1.3 to 1.8. For this analysis, a value of 1.8 is selected for *N*. The transmission coefficients can be derived along with the normalized admittance, expressed as ${\left|\tau \right|}^{2}=\frac{4}{4+Y^{2}}$. The equivalent circuit theory for the circular ring, covered by Equations (5) to (15), accounts for the interaction between the inductance and capacitance of the unit components of the circular ring. These equations facilitate a preliminary estimation of the initial physical dimensions. However, due to mutual coupling between the metals, these formulas may still contain errors. The circuit is simulated using the advanced design system (ADS), with the lumped elements subsequently optimized. The optimized values of these elements are depicted in Figure 7.

## 3. Results and Discussion

### 3.1. Simulation of ECM and Polarization Stability

**Figure 8:**
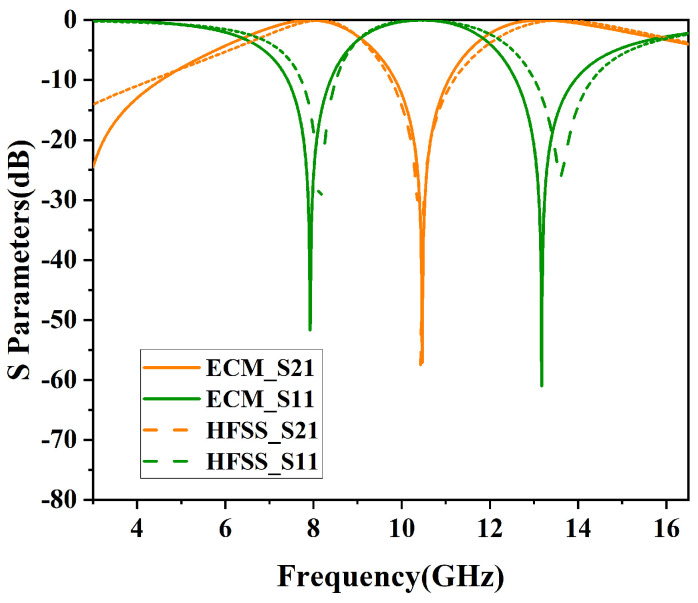
Comparison of S-parameters between ECM and HFSS.

**Figure 9:**
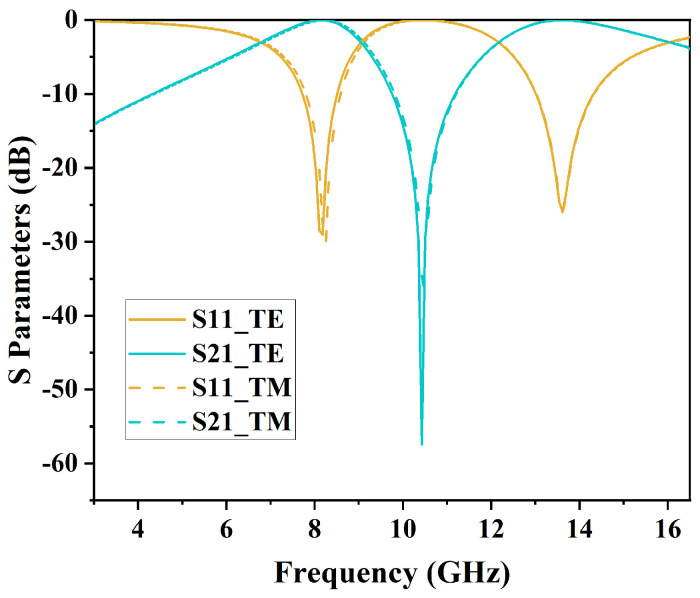
Comparison of S-parameters between TE mode and TM mode.

This design employs the software Ansys HFSS 2021 R1 to perform filtering analysis on the loaded metal unit structure, and the simulation results are compared with ECM results. Figure 8 illustrates the comparison between the simulation results and ECM results at a 0° incident angle. The ECM simulation yielded similar results to those of simulated results, which confirms the accuracy of the ECM. When the angle of incidence is at 0 degrees, the S-parameters demonstrate uniformity for both TE and TM polarizations, as shown in Figure 9.

### 3.2. Simulation of Angular Stability

**Figure 10:**
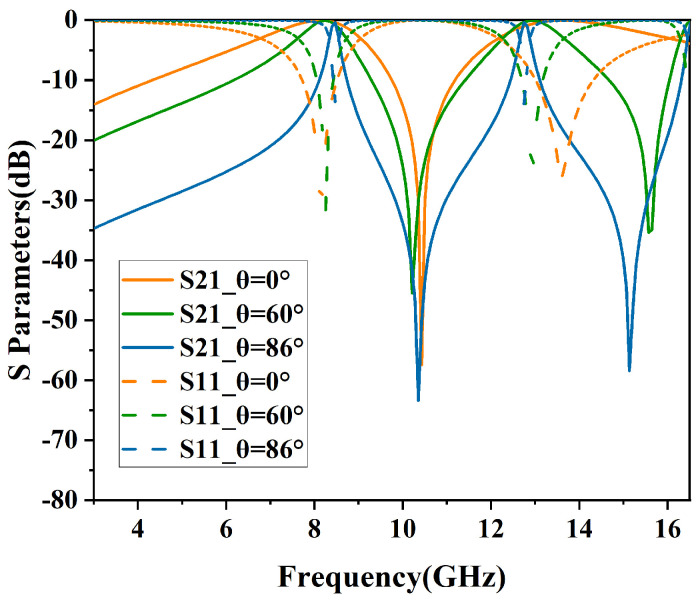
Performance of angular stability in TE mode.

**Figure 11:**
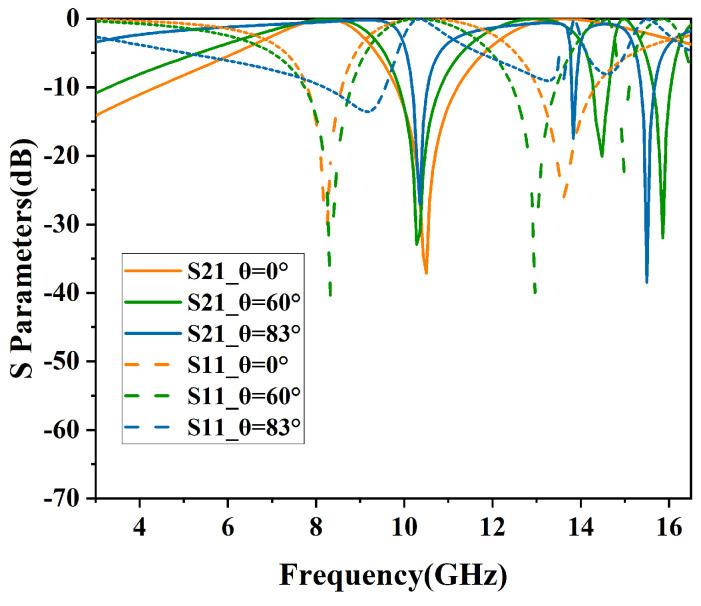
Performance of angular stability in TM mode.

The simulated results of angular stability in TE and TM polarizations are shown in Figure 10 and Figure 11. In addition, the specific performance parameters are shown in Table 2.

In Figure 10, the structure demonstrates high angular stability in TE polarization, maintaining an insertion loss of less than −1 dB for incident angles ranging from 0° to 86°. As the incident angle increases, the passband bandwidth progressively narrows. At an incidence angle of 0°, the −3 dB bandwidths ($S_{21}\geq -3\;\mathrm{dB}$) range from 6.77 GHz to 9.04 GHz and from 12.17 GHz to 16.02 GHz, with relative bandwidths of 28.6% and 27.3%, respectively.

Similarly, Figure 11 depicts the TM polarization filtering performance, where the structure maintains an insertion loss of less than −1 dB for incident angles ranging from 0° to 83°. At 0° incidence angle, the −3 dB bandwidths ($S_{21}\geq -3\;\;\mathrm{dB}$) range from 6.85 GHz to 9.11 GHz and from 12.18 GHz to 16.05 GHz, with relative bandwidths of 28.3% and 27.4%, respectively. As the incidence angle increases, the passband widths consistently increase. At 83° incidence angle, the −3 dB bandwidths ($S_{21}\geq -3\;\;\mathrm{dB}$) ranged from 3.01 GHz to 9.96 GHz and from 11.11 GHz to 13.50 GHz.

From Figure 10 and Figure 11, it can be observed that as the incident angle increases, the transmission bandwidth for TE-polarized waves gradually diminishes, whereas that for TM-polarized waves broadens. This variation in wave impedance is attributed to changes in the incident angle [15].

The equation used to determine the wave impedance for the TE mode is expressed as $Z_{TE}=\frac{Z_{0}}{\cos\theta }$, where θ represents the angle of incidence relative to the perpendicular of the FSS surface. With an increase in the incident angle, the impedance rises, leading to an increased quality factor of the load and a resultant reduction in the transmission bandwidth of the resonator. Consequently, in TE polarization, the dual-bandwidth continuously decreases. In contrast, the wave impedance for the TM mode, calculated by the formula $Z_{TM}=Z_{0}\sin\theta$, decreases with an increase in the incident angle. As a result, in TM polarization, there is a noticeable reduction in out-of-band suppression, and the bandwidth of the dual-band transmission consistently increases.

### 3.3. Surface Current and Electric Field Distribution

**Figure 12:**
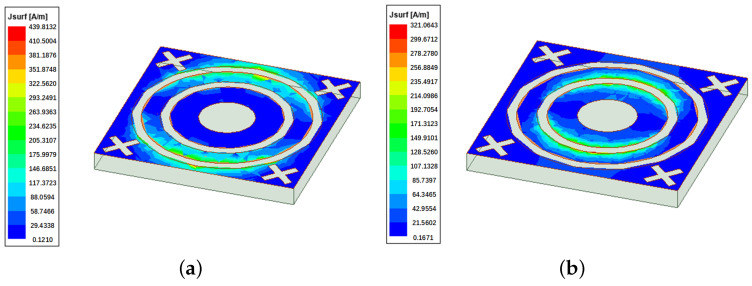
Surface current distributions: (**a**) 8.45 GHz; (**b**) 12.76 GHz.

**Figure 13:**
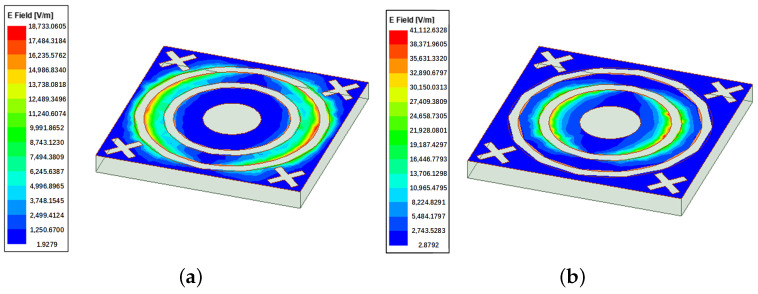
Electric field distributions: (**a**) 8.45 GHz; (**b**) 12.76 GHz.

The surface current and electric field distributions at the first and second resonant frequencies are shown in Figure 12 and Figure 13. The distribution of electric field and current is predominantly concentrated around the outer ring at the first resonance frequency of 8.45 GHz. At this frequency, there is a complementary relationship between surface current and electric field, with the electric field exhibiting greater strength while the current is relatively weaker. Conversely, at the second resonance frequency of 12.76 GHz, the current and electric field tend to disperse around the inner ring. Regions characterized by high current exhibit significant electric inductance, whereas areas with low current contribute to the capacitance of the FSS.

## 4. Fabrication and Experimental Results

**Figure 14:**
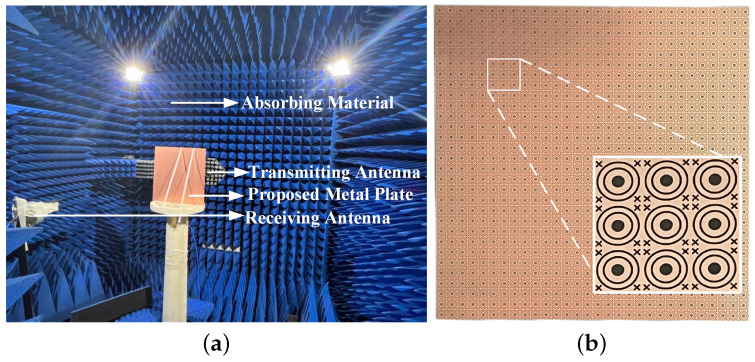
(**a**) Evaluate environment; (**b**) prototype of FSS.

To verify the simulated results, a prototype of the proposed structure is fabricated. The dimensions of the prototype are 330 mm × 330 mm with 30 × 30 elements. The measurement equipment environment is shown in Figure 14, where two sets of horn antennas are used: one operating within a frequency range of 2 GHz to 5 GHz, and the other operating within a frequency range of 5 GHz to 18 GHz. After aligning the robotic arm at a 90° angle relative to the prototype, the prototype was positioned in a vertical orientation. The structure is placed in the region from the transmitting antenna and just in front of the receiving antenna to cover the entire aperture of the receiving antenna to measure the improved transmission characteristics of the structure. The antennas are connected to a vector network analyzer (VNA) to measure the transmission coefficients of the prototype. The circular placement plate beneath the prototype was rotated at fixed angles, and successive measurements of the transmission characteristics at various angles were conducted.

**Figure 15:**
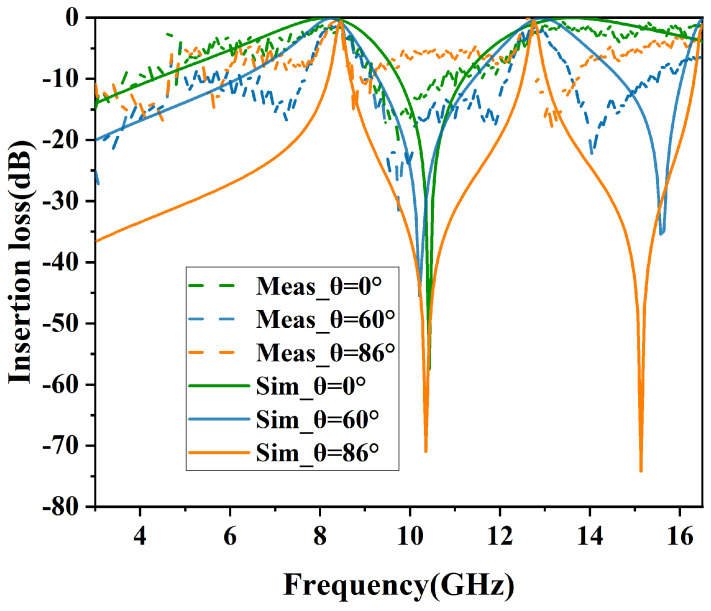
Comparison of simulated and measured results in TE mode.

**Figure 16:**
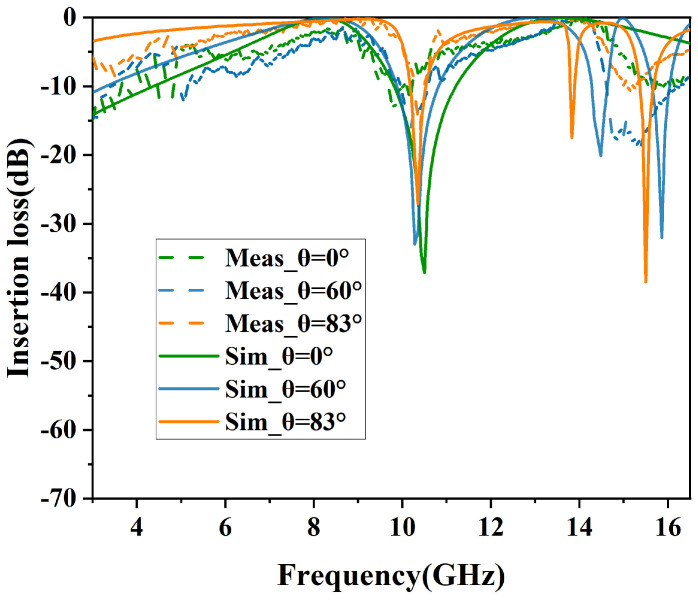
Comparison of simulated and measured results in TM mode.

The comparison of the measured results with the simulated results for TE and TM polarizations is shown in Figure 15 and Figure 16, respectively. For TE polarization, the physical model exhibits insertion losses of 0.25 dB and 0.9 dB at 0° incident angle and 3.59 dB and 4.68 dB at 86° incident angle. For TM polarization, the physical model demonstrates insertion losses of 1.07 dB and 2.9 dB at 0° incident angle and 2.88 dB and 3.05 dB at 86° incident angle. The measure results demonstrate a degree of agreement with the simulated results, consistent with the results of the experiment. However, as a result of manufacturing inaccuracies, a minor variance in the central frequency of the measured results has been observed. Moreover, a comparison of this work with previously reported structures is given in Table 3.

## 5. Conclusions

This paper proposes a dual-passband FSS characterized by superior angular stability. This structure exhibits dual-passband characteristics within the X-band and Ku-band, enabling effective operation across diverse communication scenarios. Furthermore, the center frequency point of the structure remains constant in both TE and TM polarizations, demonstrating remarkable angular and polarization stability. Specifically, the angular stability can achieve up to 86° in TE polarization and 83° in TM polarization. The consistency of the results is confirmed by the equivalent circuit method, full-wave simulation, and physical experimentation. The FSS designed in this paper has an extremely high application value in the fields of radome, multi-frequency communication, and electromagnetic shielding. It can not only improve the stealth performance of communication devices and the stability of communication systems, but also promote the integration of electronic systems.

## Figures and Tables

**Table 1:** Unit structure parameters.

Parameter	$R_{1}$	$R_{2}$	$R_{3}$	$R_{4}$	$R_{5}$	SubH
Value	5.0 mm	4.5 mm	3.0 mm	1.5 mm	6.0 mm	1.0 mm
Parameter	$W_{1}$	$W_{2}$	$W_{3}$	$W_{4}$	*P*	$L_{1}$
Value	0.5 mm	1.0 mm	0.5 mm	0.4 mm	11.0 mm	2.0 mm

**Table 2:** Insertion loss under TE/TM mode.

Angle (deg)	Insertion Loss of Center Frequency in First Passband (dB)	Insertion Loss of Center Frequency in Second Passband (dB)
0	0.36/0.24	0.98/0.98
30	0.12/0.23	0.84/0.59
60	0.71/0.08	0.30/0.10
86/83	0.76/0.52	0.70/0.99

**Table 3:** Performance comparison with existing designs.

Ref.	Type	Unit Cell	Thickness	Number of Bands	Angle (Mea.)
[8]	3D	0.21λ	0.05λ	1	60
[9]	2D	0.36λ	0.02λ	1	75
[10]	2D	0.12λ	0.05λ	1	80
[13]	2D	0.17λ	0.04λ	1	85
[16]	2.5D	0.21λ	0.11λ	1	60
[18]	3D	0.33λ	0.34λ	1	45
[19]	2D	0.08λ	0.01λ	2	60
[20]	2D	0.27λ	0.49λ	2	60
[15]	2D	0.15λ	N.A.	2	60
This work	2D	0.31λ	0.02λ	2	86/83

## Data Availability

Data are contained with in the article.
